# From morphology to biochemical state – intravital multiphoton fluorescence lifetime imaging of inflamed human skin

**DOI:** 10.1038/srep22789

**Published:** 2016-03-23

**Authors:** Volker Huck, Christian Gorzelanny, Kai Thomas, Valentina Getova, Verena Niemeyer, Katharina Zens, Tim R. Unnerstall, Julia S. Feger, Mohammad A. Fallah, Dieter Metze, Sonja Ständer, Thomas A. Luger, Karsten Koenig, Christian Mess, Stefan W. Schneider

**Affiliations:** 1 Heidelberg University, Medical Faculty Mannheim, Experimental Dermatology, Theodor-Kutzer-Ufer 1-3, 68167 Mannheim, Germany; 2 University of Münster, Department of Dermatology, Von-Esmarch-Str. 58, 48149 Münster, Germany; 3 University of Konstanz, Department of Biophysical Chemistry, Universitätsstr. 10, 78457 Konstanz, Germany; 4 Saarland University, Mechatronics & Physics, Campus A5 1, 66123 Saarbrücken, Germany; 5 JenLab GmbH, Schillerstr. 1, 07745 Jena, Germany

## Abstract

The application of multiphoton microscopy in the field of biomedical research and advanced diagnostics promises unique insights into the pathophysiology of inflammatory skin diseases. In the present study, we combined multiphoton-based intravital tomography (MPT) and fluorescence lifetime imaging (MPT-FLIM) within the scope of a clinical trial of atopic dermatitis with the aim of providing personalised data on the aetiopathology of inflammation in a non-invasive manner at patients’ bedsides. These ‘optical biopsies’ generated via MPT were morphologically analysed and aligned with classical skin histology. Because of its subcellular resolution, MPT provided evidence of a redistribution of mitochondria in keratinocytes, indicating an altered cellular metabolism. Two independent morphometric algorithms reliably showed an even distribution in healthy skin and a perinuclear accumulation in inflamed skin. Moreover, using MPT-FLIM, detection of the onset and progression of inflammatory processes could be achieved. In conclusion, the change in the distribution of mitochondria upon inflammation and the verification of an altered cellular metabolism facilitate a better understanding of inflammatory skin diseases and may permit early diagnosis and therapy.

Atopic dermatitis (AD) is a highly prevalent inflammatory skin disease with increasing incidence, mainly in developed countries. Manifestation of AD usually occurs in early childhood and is often followed by the development of allergies and asthma. According to current data, approximately 10–30% of newborns are affected by AD^1^. The underlying pathophysiological mechanisms of the disease are under debate, and a complex interplay between genetic, epigenetic and environmental factors is suggested^2^. However, further knowledge of the pathophysiology of AD is essential to improve the present therapeutic options.

To overcome the limitations of current mouse models and the restricted ability to analyse the skin of human patients via invasive techniques such as biopsies, we have applied multiphoton-based intravital tomography (MPT) equipped with a spectral fluorescence lifetime imaging module^3^ (MPT-FLIM) for the non-invasive *in vivo* analysis of human skin. Currently, the diagnosis of skin diseases is mainly based on the skills of the dermatologist or on the histological analysis of biopsies (the current gold standard). Physical examination of the patient is limited to the macroscopic surface level, and taking biopsies is an invasive approach, resulting in the formation of scars, thus excluding a longitudinal analysis of specific skin lesions. In contrast to alternative *in vivo* techniques such as ultrasound or confocal laser scanning microscopy^4^, MPT-FLIM allows subcellular *in vivo* imaging of thus far unknown parameters of inflammation. Therefore, the aim of the present study was to validate multiphoton-based tomography as a unique non-invasive tool for the morphological and biochemical characterisation of human skin.

Five-dimensional MPT-FLIM analysis comprises spatial (first to third dimension) and spectrally resolved fluorescence lifetime imaging (fourth and fifth dimension) by means of femtosecond laser pulses. This laser technology allows deep penetration of light into the skin and therefore the visualisation of the epidermis as well as the upper part of the dermis with subcellular resolution. *In vivo* investigations in humans exclude the application of fluorescence labelling of cells or proteins but are open to the use of naturally occurring fluorophores, such as melanin, keratin or NADH^5^,^6^. Multiphoton excitation of these endogenous fluorophores therefore enables the non-invasive high-resolution examination of human skin without damaging the surrounding tissue^7^.

Previous *in vitro* studies applying multiphoton microscopy showed a link between the pro-inflammatory activation of macrophages and the NADH signal intensity, suggesting an increased production of reactive oxygen species and a rearrangement of cellular metabolism^8^. To determine the cellular metabolism of epidermal cells *in vivo*, we utilised MPT-FLIM, an approach that enables an excitation energy independent readout of the NADH status^9^,^10^. *In vivo* FLIM measurement of NADH has been used in the context of skin cancer diagnoses in rodents^11^ and was recently successfully applied in the clinic^12^,^13^,^14^. The FLIM signal depends strongly on the microenvironment of the excited substance, enabling the discrimination of protein-bound and free NADH^15^. Free NADH was found to exhibit a fluorescence lifetime between 200 and 450 ps, whereas protein-bound NADH exhibits a prolonged lifetime in the range of 2,000 to 3,000 ps^16^,^17^. The ratio of free to protein-bound NADH is reflected by the mean fluorescence lifetime (tau_m_)^18^, which serves as an intravital readout of the cellular metabolic state^19^.

Our clinical trial comprised 45 patients and followed their individual inflammatory progression over three months. For the first time, we established MPT-FLIM as a reliable and valuable tool for the clinical investigation of inflammatory skin diseases, facilitating a rapid, cost-efficient, non-invasive *in vivo* examination for diagnostic and therapeutic purposes in humans.

## Results

### Alignment of intravital multiphoton tomographic data with classical skin biopsy analysis

**Figure 1:**
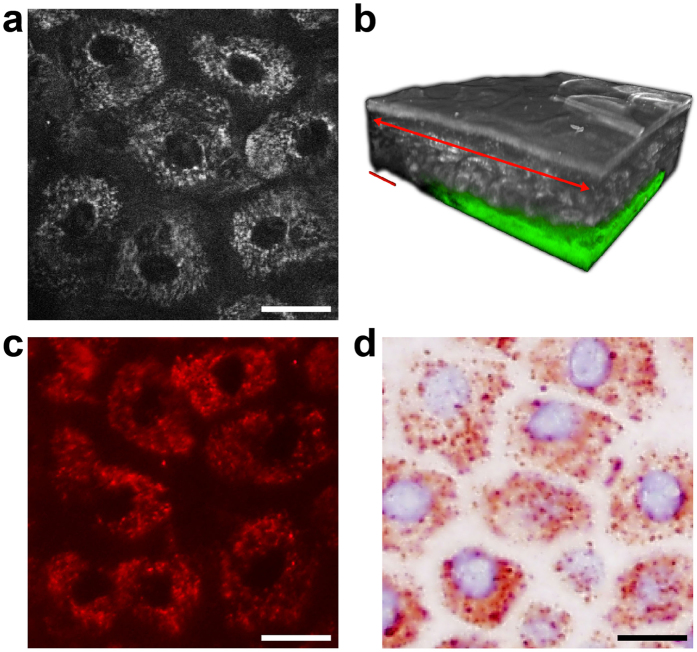
Multiphoton tomographic optical biopsy and mitochondria staining of healthy human skin. (**a**) A representative multiphoton optical section of the *Stratum granulosum* of healthy skin *in vivo*. (**b**) A multiphoton tomographic three-dimensional reconstructed skin cube. The examined target section of (**a**) is indicated (red arrow), and dermal collagen in the *Stratum papillare* is pseudocolour-coded in green. Alignment with mitochondria-specific (**c**) immunofluorescence staining and (**d**) immunohistochemistry of a corresponding skin region in the *Stratum granulosum* performed *ex vivo*. Scale bars correspond to 20 μm.

**Figure 2:**
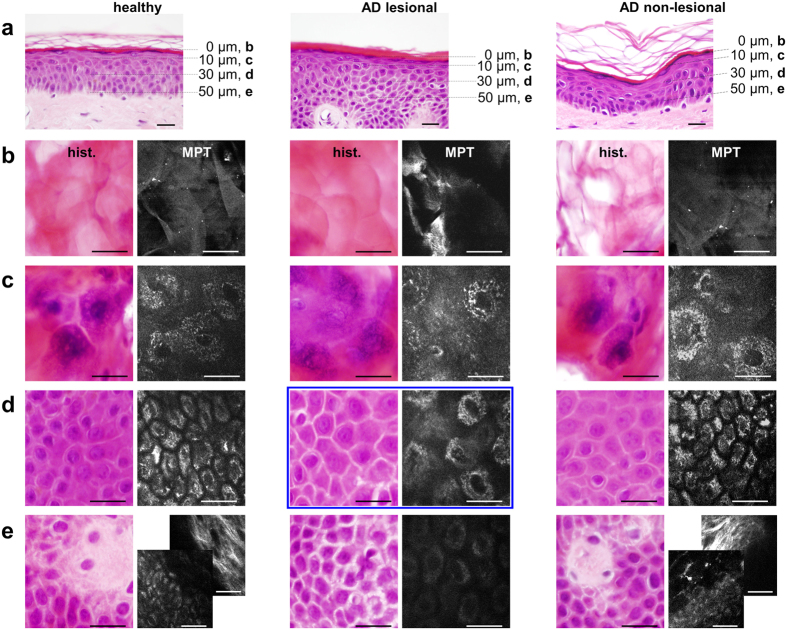
Gold standard alignment of intravital multiphoton tomography. (**a**) Standard vertical histological sections of the skin from healthy subjects (healthy), the ostensibly healthy skin of a patient affected by AD (AD non-lesional) and lesional atopic skin (AD lesional) examined via multiphoton tomography. Alignment of horizontal histological sections (‘hist.’-column) with multiphoton tomographic sections (‘MPT’-column) of the same regions of healthy, AD non-lesional and AD lesional areas of skin at distinct skin depths of 0 μm (*Stratum corneum* (**b**)), 10 μm (upper *Stratum granulosum* (**c**)), 30 μm (*Stratum spinosum* (**d**)) and 50 μm (**e**), representing the interface between the epidermal *Stratum basale* and the *Stratum papillare* of the dermis. A comparison of ‘hist.’ with ‘MPT’ illustrates the identical validity of assessing the morphological skin architecture and detecting characteristic skin morphologies. In the ‘AD lesional’ patient, the dermis is not accessible at a depth of 50 μm due to typical thickening of the epidermis. The accessibility of the dermis was demonstrated through additional imaging of the collagen-specific generation of second harmonics, as shown in the backmost image of the composite ((**e**) ‘MPT healthy’ and ‘MPT AD non-lesional’). Furthermore, the keratinocytes appear to be dispersed by inflammatory intercellular oedema. At a depth of 30 μm ((**d**) ‘AD lesional’), MPT is conspicuously superiour to the histological preparation regarding the assessment of the intensity of intercellular oedema (marked with a blue box). Scale bars correspond to 20 μm.

We used MPT as an artefact-free, painless approach that provides an ‘optical biopsy’ with subcellular resolution in human skin (Fig. 1a, see also Supplementary Movie S3). Previous studies of multiphoton imaging have suggested that NADH is the major natural fluorophore in human cells^20^,^21^,^22^. The bright spots observed in the cytoplasm of keratinocytes surrounding dark nuclei when using this technique (Fig. 1b) are mainly due to multiphoton-excited NADH, ensured by the combination of the utilised excitation wavelength and spectral emission filtering (see Methods section). These spots are located within the mitochondria as shown by immunohistochemical and fluorescence microscopic alignment (Fig. 1). Based on these settings, we compared the same skin region in healthy subjects and patients affected by AD via MPT and subsequently via histological analyses (Fig. 2). It must be emphasised that MPT allows horizontal sectioning of the skin, and we therefore also collected comparative biopsies cut in a horizontal manner. Each subject was clinically examined by a dermatologist and by MPT over a period of three months. During each session, we analysed one lesional (inflamed tissue) and one non-lesional (ostensibly healthy) skin region in comparison to the areas in age-correlated healthy subjects. As shown in Fig. 2, the analyses of histological biopsies and multiphoton-based intravital tomographic images of lesional skin areas were of identical pathognomonic validity (compare ‘hist.’-columns and skin depth correlated ‘MPT’-columns, Fig. 2b–e). Upon MPT, we were able to detect the characteristic skin morphologies of AD, such as intercellular oedema and an impaired architecture, accompanied by thickening of the epidermis in lesional skin. In particular, due to the absence of artefact-inducing embedding, dehydration and staining procedures, the MPT technique is markedly superior regarding assessment of the intensity of intercellular oedema (Fig. 2d, AD lesional, marked with a blue box).

### Detailed morphological analysis of the cellular mitochondrial distribution

**Figure 3:**
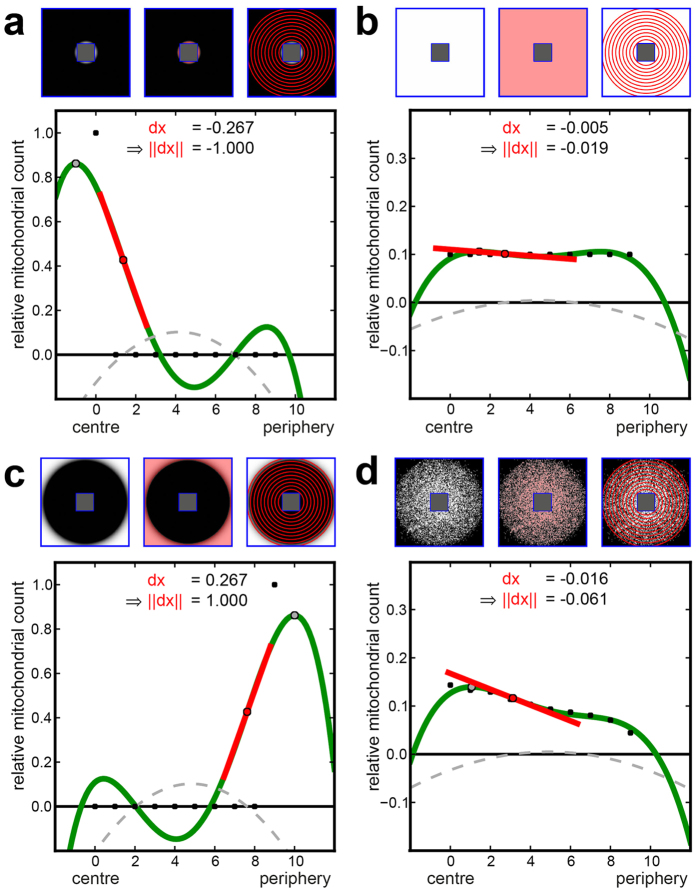
Annuli filling analysis (AFA) of generated dummy cells. (**a**–**d**) Upper row: Quadratic dummy cells with nuclear (central rectangle) and cell membranes marked in blue; the cytoplasm lying in between is the used 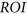 for AFA. Upper left: An intensity image of the mitochondrial signal within the 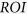. Upper centre: Mitochondrial pixels marked with a light red overlay. Upper right: Circular regions (annuli) from the centre to the periphery (A_0_, …, A_9_) are visualised as red concentric circles. Graph: A scatterplot of the relative mitochondrial count m_0_, …, m_9_ per annulus (black dots) is shown. The interpolated polynomial is shown in green, the inflexion point as a red dot, the tangent at the inflexion point as a red line, the dominant maximum as a grey dot, and the second derivative of the polynomial as a dashed grey curve. (**a**) All mitochondrial pixels reside within A_0_, resulting in maximal centralisation and, thus, a minimal (normed) inflexion point gradient (dx = −0.267, ||dx|| = −1.0). (**b**) A homogeneous distribution over all annuli is shown. (**c**) All mitochondrial pixels are located within A_9_, resulting in maximal peripheralisation. (**d**) A cell-like even distribution results in slight centralisation.

**Figure 4:**
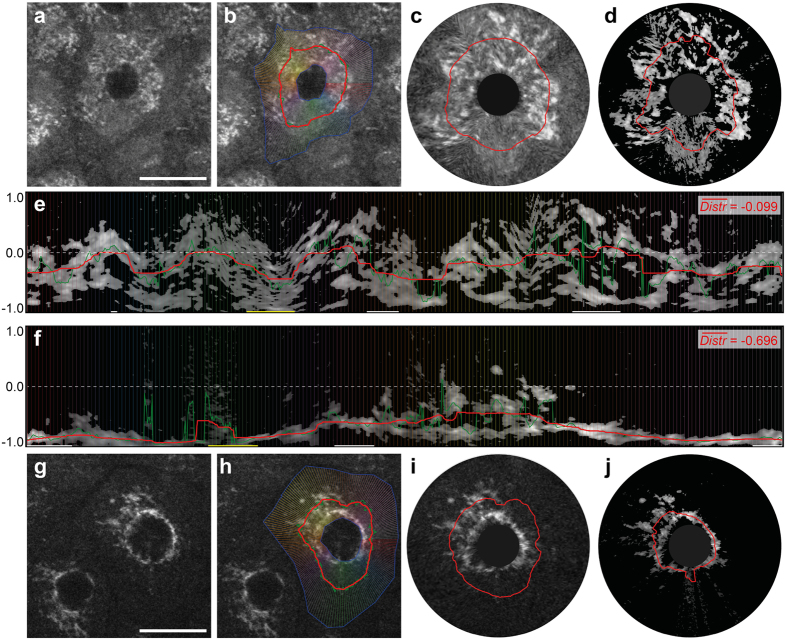
Radial profiles analysis (RPA) of human epidermal cells of the *Stratum granulosum*. Archetypical keratinocytes of the *Stratum granulosum* in healthy (**a–e**) and lesional (**f–j**) skin areas have been selected. (**a,g**) Representative multiphoton tomographic cell images. (**b,h**) The cytoplasm bounded by the blue polygons is used as 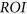 for RPA. Radial profile lines (coloured) and plots of mitochondrial distribution values (green: raw; red: smoothed) are shown. Virtual cell reconstructions based on radial profiles with smoothed distribution values are depicted before (**c,i**) and after thresholding (**d,j**). Consequently, the thresholded radial profiles for healthy (**e**) and lesional (**f**) cells are displayed with the calculated mean distribution values (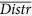). Outliers are marked with horizontal bars on the x-axis (yellow: short, white: long). Scale bars correspond to 20 μm.

In addition to pathognomonic skin morphologies, we found a strikingly altered multiphoton tomographic pattern within single keratinocytes. Upon inflammation, the subcellular mitochondrial distribution (MD) appeared to be affected. In lesional skin, the mitochondrial signal was apparently stronger in the vicinity of the nuclei compared with an almost homogenous arrangement in healthy skin areas. For a detailed quantification of MD, we developed two independent computational algorithms: annuli filling analysis (AFA) and radial profiles analysis (RPA). Briefly, in AFA the signal intensity is plotted against the distance to an artificial nucleus from the centre to the periphery. As an appropriate indicator of MD characteristics, the normalised gradient (||dx||) at the dominant inflexion point ranges from −1.0 (maximal centralisation, Fig. 3a) across 0.0 (even signal distribution, Fig. 3b) to 1.0 (maximal peripheralisation, Fig. 3c). Owing to the potential limitations of AFA for irregularly shaped cells or eccentric nuclei, we introduced RPA, in which the mitochondrial signal is sampled on equiangular spoked lines (Fig. 4b,h) within the cytoplasm. Here, MD characteristics are quantified based on the RPA distribution value (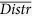): In contrast to the even signal distribution found in healthy cells (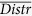 ≈ 0.0, Fig. 4a–e), lesional cells exhibit centralisation, resulting in a significant decrease of 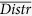 (Fig. 4f–j). For a detailed description of both approaches, please see also the Methods section.

**Figure 5:**
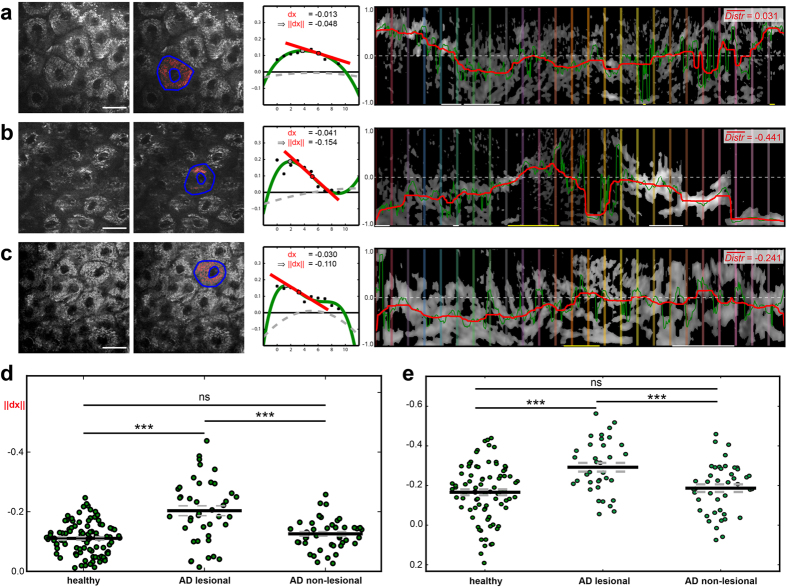
Mitochondrial distribution of patients’ epidermal cells calculated via AFA and RPA. (**a**) Within a typical *Stratum granulosum* cell layer in healthy skin (left image), the selected cell marked in blue (second image) shows a homogenous mitochondrial distribution based on AFA, corresponding to a small gradient at the inflexion point (third image). Accordingly, RPA indicates a 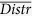 value near zero (right image). (**b**) The selected cell from inflamed skin (AD lesional) shows mitochondrial centralisation around the nucleus, resulting in a decreased inflexion point gradient and 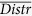 value compared with (**a**).(**c**) The mitochondria of the non-lesional cell show slight centralisation. (**d**) An AFA comparison of relative gradients (||dx||) of cells from healthy, lesional and non-lesional subjects. (**e**) An RPA 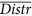 plot of cells from healthy, lesional and non-lesional patients. Each green point represents one segmented cell. Scale bars correspond to 20 μm.

In applying these models to the entire MPT dataset from the clinical trial, we focused on the outermost layer of living epidermal cells (*Stratum granulosum*) and observed even partitioning of the mitochondria in healthy skin, as expected (Fig. 5a). By contrast, the MD within inflamed (AD lesional) skin showed perinuclear accumulation, as quantified by a significant negative shift of both ||dx|| and 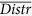 (Fig. 5b), whereas the MD in non-lesional skin (Fig. 5c) was comparable to that of healthy skin. The statistical analysis of MD in the *Stratum granulosum* of all subjects included in the clinical trial is shown in Fig. 5d,e.

### *In vitro* characterisation of the energy status of human keratinocytes using MPT-FLIM

**Figure 6:**
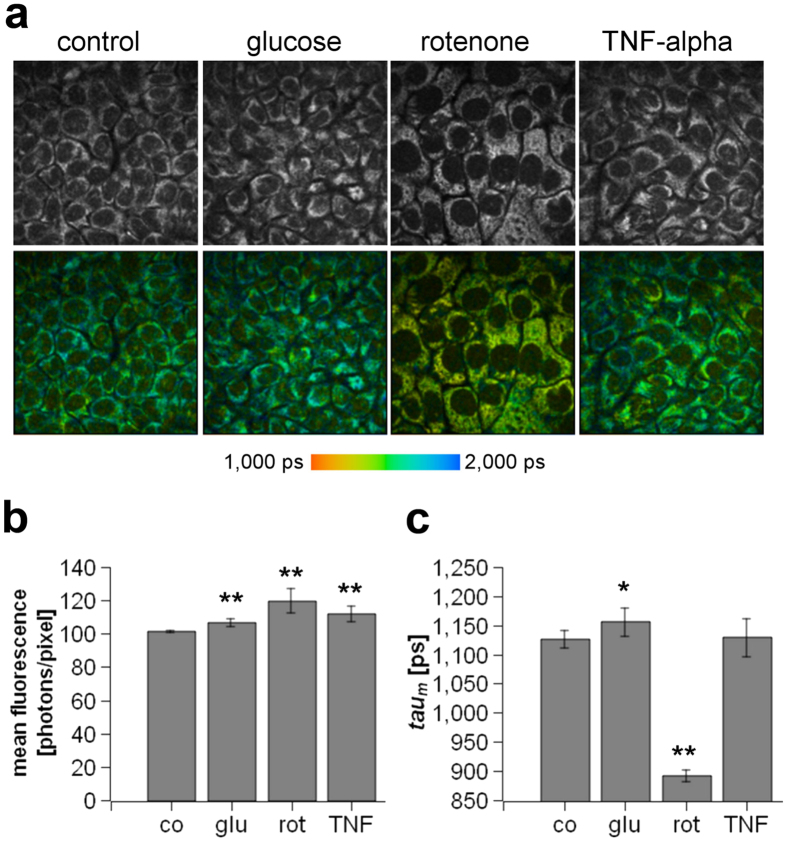
Evaluation of the energy status of human keratinocytes *in vitro*. (**a**) Fluorescence intensity images reflecting the total amount of cellular NADH (first row) and fluorescence lifetime images (FLIM) (second row) of non-treated keratinocytes (control) or keratinocytes treated either with 20 mM glucose, 5 μM rotenone or 10 nM TNF-alpha. The colour-coded bar reflects the fluorescence lifetime, ranging from 1,000 ps (red) to 2,000 ps (blue). (**b**) Quantification of the mean fluorescence intensity of non-treated keratinocytes (co) or keratinocytes treated with glucose (glu), rotenone (rot), or TNF-alpha (TNF). (**c**) Quantification of the mean fluorescence lifetime (tau_m_) of non-treated keratinocytes (co) or keratinocytes treated with glucose (glu), rotenone (rot), or TNF-alpha (TNF). *P < 0.05, **P < 0.01.

Because of the inflammation-correlated morphological alterations and shift of MD we observed *in vivo* (see Fig. 5d,e), we additionally aimed to demonstrate that MPT-FLIM is a suitable tool for the interpretation of cellular metabolism in human keratinocytes exposed to distinct stimuli that are known to alter their metabolic state *in vitro*. The molecular basis of skin inflammation is neither limited to keratinocytes nor to single stimuli, thus preventing the establishment of an accurate *in vitro* system. However, to allow a general understanding of the NADH metabolism of keratinocytes to be obtained under inflammatory conditions, we treated human keratinocytes *in vitro* with the pro-inflammatory cytokine TNF-alpha. For comparison, we manipulated the cellular metabolism of keratinocytes by adding glucose or rotenone. Representative images of the fluorescence intensity images (first row) and the corresponding fluorescence lifetime images (second row) are presented in Fig. 6a. Most of the fluorescence signals were localised in the cytoplasm and were related to mitochondrial NADH (see also Fig. 1)^23^. An increase of the fluorescence intensity was measured in glucose-, TNF-alpha- and rotenone-treated keratinocytes (Fig. 6b). The obtained tau_m_ values are depicted in Fig. 6c. A massively increased amount of free NADH was apparent in rotenone-treated keratinocytes, as indicated by a reduction of the tau_m_ value. In glucose-treated keratinocytes, an increase of tau_m_ was found, suggesting a greater amount of protein-bound NADH.

### MPT-FLIM provides evidence of inflammation-related alteration of the cellular metabolism

**Figure 7:**
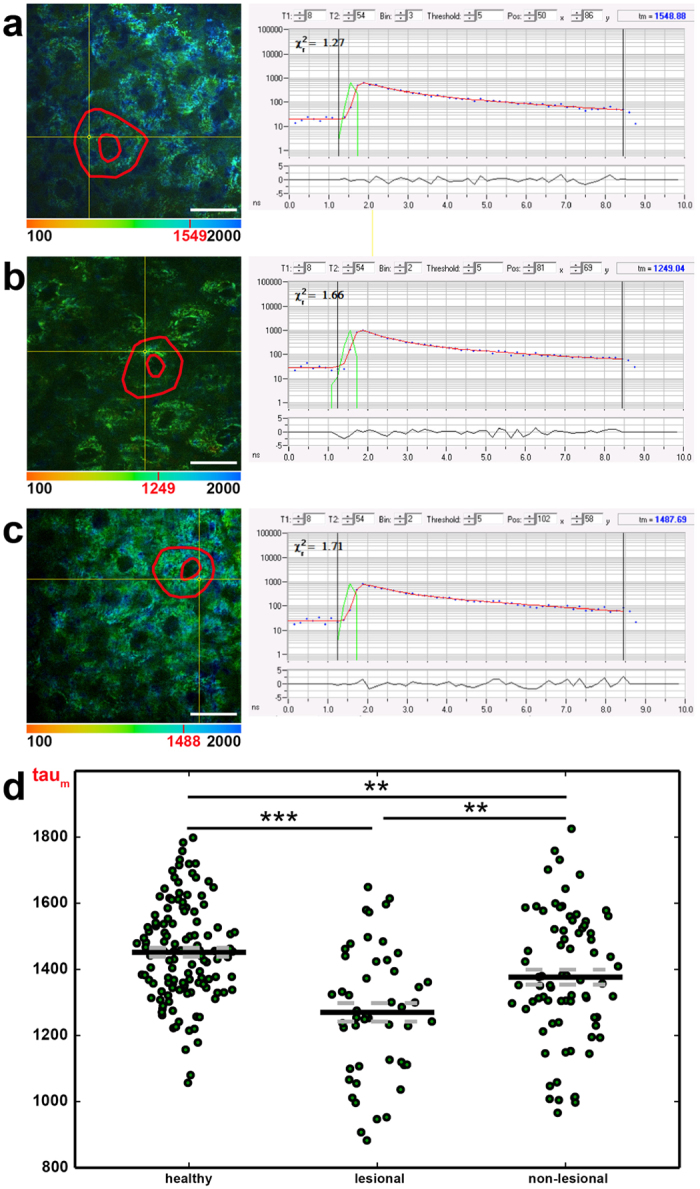
Comparison of the fluorescence lifetime (tau_m_) of healthy, lesional and non-lesional *Stratum granulosum* cells. Overlay images of MPT morphology and colour-coded tau_m_ values of representative healthy (**a**), lesional (**b**) and non-lesional (**c**) cell layers, with one segmented cell contoured in red. A low tau_m_ (red = 100 ps) is visualised with warm colours and a high tau_m_ (blue = 2,000 ps) with cool colours. The subsequent graphs show the FLIM curve of one selected pixel (yellow crosshairs); its tau_m_ value is denoted in red in the colour bar. (**d**) The distribution of tau_m_ in healthy, lesional and non-lesional cell layers of patients is displayed. Each green point represents the mean tau_m_ value of one image. Scale bars correspond to 20 μm.

To investigate whether the altered subcellular localisation of mitochondria is associated with an altered cellular metabolism *in vivo*, we applied MPT-FLIM in the setting of the clinical trial. We calculated the mean tau_m_, reflecting the ratio of free-to-protein-bound NADH, for all images of the *Stratum granulosum* obtained for the entire study population. This FLIM analysis, shown in Fig. 7, revealed a significant decrease of tau_m_ in inflamed skin compared with healthy skin (1,270 +/− 28 ps to 1,452 +/− 13 ps, Fig. 7d). In contrast to the histological and MPT morphological analyses, the MPT-FLIM analysis allowed distinction between the healthy skin and non-lesional skin of AD-affected patients (1,452 +/− 13 ps to 1,377 +/− 22 ps, Fig. 7d), indicating a shift of the metabolic state.

**Figure 8:**
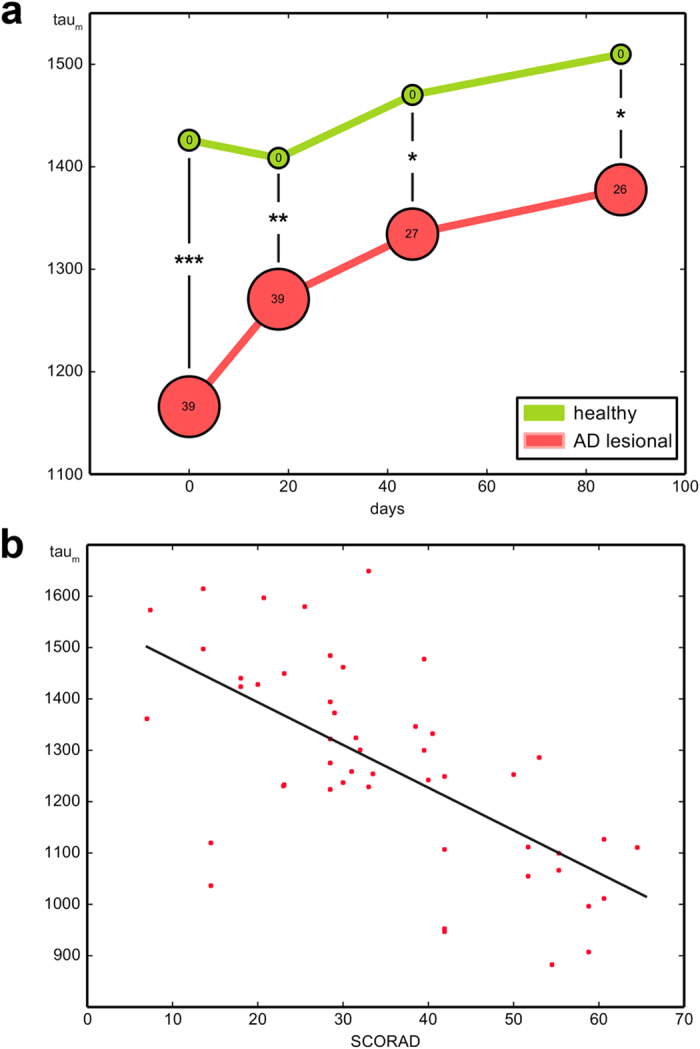
Overall fluorescence lifetime (tau_m_) in the course of the clinical trial. (**a**) Mean tau_m_ values of lesional skin areas in all patients suffering from AD continuously show a significant difference in the tau_m_ of healthy skin over the entire term. The bubble size represents the disease severity measured via SCORAD. (**b**) Plotting the tau_m_ of lesional skin against the corresponding inflammatory state measured via SCORAD, a significant linear correlation is revealed: tau_m_ decreases with increasing SCORAD.

While the mean inflammatory state of patient skin slightly decreased during the course of the study (as indicated by a decreasing *Severity Score of Atopic Dermatitis* (SCORAD)), we could detect an increase of tau_m_ in lesional skin areas from 1,166 +/− 40 ps to 1,378 +/− 45 ps. Moreover, we observed a continuous significant difference in the measures throughout the study compared with the tau_m_ of healthy skin (Fig. 8a). Plotting the tau_m_ values of lesional skin against the corresponding inflammatory state measured via SCORAD, we found a significant linear correlation, in which tau_m_ decreased with an increasing SCORAD (PCC 0.65, p < 0.0001, Fig. 8b).

To exclude systemic shifts influencing our MPT-FLIM measurements, we plotted tau_m_ as a function of the day and month of examination. As expected, in this nonsense correlation, the examination day did not show any impact on tau_m_ (see Supplementary Fig. S1).

## Discussion

The present data demonstrate the translation of a medical assessment to *in vivo* MPT-FLIM and morphometric analyses in the context of a clinical setting for the first time. We investigated morphological alterations as well as the metabolic status of keratinocytes in the background of AD, an inflammatory skin disease with a complex pathophysiology and a high prevalence. In line with classical histological analyses, we were able to detect the formation of intercellular oedema and thickening of the epidermis that correlated with the severity of inflammation. Moreover, in the objective computational analysis of the morphological dataset, MPT provided information on the reorganisation of mitochondria. Two independent morphometric algorithms reliably showed an even MD in healthy skin and a perinuclear accumulation in inflamed skin (Fig. 5).

As this observation suggested a direct metabolic impact, we first performed *in vitro* MPT-FLIM examinations of a human keratinocyte cell line upon stimulation with various agents known to alter keratinocyte metabolism (Fig. 6). Based on our data, a decrease of the mean fluorescence lifetime (tau_m_) indicated an accumulation of free NADH in the mitochondria, suggesting an overload of the respiratory electron transport. This overload could be related to either a lack of oxygen or hyperproduction of NADH during glycolysis. Malfunction of the mitochondrial electron transport chain is a marker of cellular stress such as apoptosis and is accompanied by the production of reactive oxygen species^24^.

In line with these *in vitro* findings, we were able to measure a comparable change of cellular metabolism in the inflamed skin of patients using MPT-FLIM (Fig. 7). Additionally, the decrease of tau_m_ correlated with the severity of inflammation (Fig. 8b), pointing towards increased stress in keratinocytes within the affected skin regions.

Bridging the gap between the physiological basis of the cellular metabolism and its potential impact on a patient’s pathogenesis, this study demonstrates that multiphoton tomography equipped with a spectral FLIM module might be successfully used as a diagnostic tool in the field of skin inflammation research. In contrast to the exclusive morphological analysis of inflamed skin, MPT-FLIM is able to non-invasively demonstrate that even the apparently healthy (non-lesional) skin of patients affected by AD exhibits pathological features resembling those of eczematous skin lesions. Therefore, our data support previous genetic, histological and molecular biological findings from non-lesional skin regions^25^,^26^,^27^.

In contrast to classical examination tools, the non-invasive nature of MPT-FLIM might help to identify regions with nearly subclinical inflammation. Clear identification of these skin regions will be beneficial to initiate early proactive treatment, preventing inflammatory exacerbation. As a unique feature, MPT-FLIM combined with standardised computational analysis generates new *in vivo* parameters that can be used to detect and monitor pathophysiological alterations in the earliest state of inflammatory processes in human skin, prior to clinical manifestation. Thus, the *in vivo* application of MPT-FLIM offers the possibility of directly examining the pathogenesis of inflammatory skin diseases, in addition to consecutive observation of individual aetiopathology in a non-invasive manner.

## Methods

### Multiphoton tomography

In the present study, we applied a certified CE-marked five-dimensional intravital multiphoton tomographic system (MPT). The technical setup, based on a *DermaInspect* multiphoton microscope, was established within the framework of the BMBF project ‘5D-IVT’ by JenLab (JenLab GmbH, Jena, Germany). Near-infrared laser pulses with a pulse length of 100 fs provided by a Titanium:Sapphire tuneable laser system (Mai Tai, Newport Spectra-Physics, Santa Clara, CA, USA) were used for the excitation of endogenous fluorophores in human skin. The excitation laser beam was attenuated by the use of a Glan calcite polariser and scanned by two galvanometric mirrors. After passing a beam expander and collimator, the laser pulses are reflected by a dichroic beam splitter into a 40x oil immersion microscope objective with a numerical aperture of 1.3 (Carl Zeiss Jena GmbH, Jena, Germany).

The emitted fluorescence light was transmitted and cleared by an additional shortwave-pass filter (F37-490 BrightLine HC 490/LP and F75-680 Multiphoton-Emitter HC 680/SP, F39-461 BrightLine HC 460/60, F39-390 BrightLine HC 390/40; Semrock Inc., Rochester, NY, USA) and was split into three distinct spectral regions by a set of dichroic beam splitters (F43-031 425DCXR, F33-499 495DCXR; Chroma Technology Corp., Bellows Falls, VT, USA).

Three separate photomultipliers (PMTs) provided intensity images of the sample, and each PMT was readout via time-correlated single photon counting to calculate wavelength-range-specific fluorescence lifetime values (MPT-FLIM). The PMT data were processed by a high-resolution time-correlated single photon counting imaging module (SPC 830, Becker & Hickl GmbH, Berlin, Germany).

This setup allows us to non-invasively excite fluorophores in human skin *in vivo* to create high-resolution autofluorescence images and fluorescence lifetime images with a penetration depth of 150 μm. An excitation wavelength of 710 nm and an emission bandpass filter (460/60 nm) were used to match the spectrum of NADH.

### Study design

Twenty healthy volunteers and 25 patients affected by AD who were undergoing basic therapy were enrolled in this study. For each subject, two different skin areas were analysed during four clinical visits over three months to obtain a survey of the diverse states of inflammation. In each visit, a physician diagnosed the state of inflammation according to the SCORAD^28^, and a local score of inflammatory manifestation in the investigated skin areas was assigned via clinical inspection. To offer the patients the most comfortable examination, we chose one lesional and one non-lesional skin area of the volar forearm. The targeted skin area was directly coupled to MPT using an *in vivo* adapter provided by JenLab. Sequences of confocal sections with 10 μm penetration steps, an edge length of 100 μm and an exposure time of 25 s per image were recorded, starting at the outmost epidermal layer (*Stratum corneum*), defined as a depth of 0 μm, and ending at the papillary dermis, at approximately 150 μm. The study was conducted conforming to the guidelines of the *Declaration of Helsinki*^29^ and *The International Conference on Harmonisation of Technical Requirements for Registration of Pharmaceuticals for Human Use (ICH)*^30^. It was approved by the *Bundesinstitut für Arzneimittel und Medizinprodukte (BfArM)* of Germany and the Ethics Committee of the *Medical Association Westfalen-Lippe* (Münster, Germany). Appropriate informed consent was obtained from all subjects.

### Histological alignment

Immediately after the last clinical visit, punch biopsies were taken from target skin areas in three healthy subjects and three affected patients. The biopsies were split for standard vertical and horizontal histological preparation and stained with haematoxylin-eosin, for classical histological examination, or with the anti-mitochondrial antibody MTC02 ab3298 (Abcam, Cambridge, MA, USA), for alignment of the mitochondrial distribution, and were examined by a histopathologist and aligned to the corresponding MPT images.

### Data preparation and analysis of the cellular mitochondrial distribution

#### Cell segmentation

Because the mitochondrial distribution is defined per cell, the cytoplasm of different typical cells must be segmented in a preceding step. This segmentation is performed manually by defining (Software MIPAV^31^) two non-self-intersecting polygons: an inner polygon *P*_*nucleus*_ tracing the nucleus and an outer polygon *P*_*cell*_ tracing the cell membrane. The cytoplasm polygon is then defined by the difference *P*_*cytoplasm*_ = *P*_*cell*_ − *P*_*nucleus*_, which is the region of interest (*ROI*) for the subsequent steps. To correct irregular illumination, we used a previously reported Gaussian filtering-based method (see Supplementary Methods and Supplementary Fig. S2)^32^,^33^.

#### Mitochondrial pixel classification

The mitochondrial regions of the cytoplasm are defined by a strong NADH signal resulting in a high photon count at the corresponding positions. The shading corrected photon count image *I*_*corr*_ used for the quantitative analysis of MD consists of a 128 × 128 matrix and each pixel *I*(*x*, *y*) contains the number of detected photons. To identify all pixels 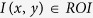 representing mitochondrial areas, an automatic thresholding algorithm is used^34^. The algorithm assumes that *I*_*corr*_ consists of two classes, foreground (high intensity) and background (low intensity) pixels, and iteratively calculates the optimal threshold by minimising the variance within each class. The detected mitochondrial pixels are coloured red in Figs 3 and 5.

#### Annuli filling analysis* (AFA)*

To measure the spatial distribution of mitochondrial pixels within the cytoplasm of each cell, the 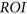 is divided into 10 circular regions (annuli) from the centre to the periphery (*A*_0_, …, *A*_9_) defined by concentric circles *C*_0_, …, *C*_9_ (see upper right of Fig. 3a–d). With 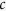 as the centroid of the inner polygon *P*_*nucleus*_, the radius *r*_0_ of the innermost circle *C*_0_ is defined by the maximum distance between *c* and the bounding box of *P*_*nucleus*_. The radius *r*_9_ of the outer circle *C*_9_ is the minimum distance between 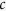 and the bounding box of *P*_*cell*_. The radii *r*_1_, …, *r*_8_ are defined by 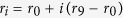/9 with 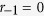. Given the Euclidian distance 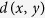, a pixel 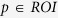 resides in annulus 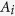 if 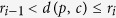. Following this scheme, every mitochondrial pixel 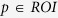 can be assigned to exactly one annulus *A*_*i*_, and a relative mitochondrial count *m*_*i*_ can be calculated per *A*_*i*_ by dividing the number of mitochondrial pixels by the number of all pixels in *A*_*i*_.

In the next step, the relative mitochondrial counts *m*_0_, …, *m*_9_ (black points in the graphs of Figs 3 and 5) are used to calculate a 4^th^ degree polynomial 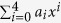 via weighted polynomial interpolation (green plot in the graphs of Figs 3 and 5). Two extra points 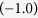 and 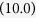 (not shown in Figs 3 and 5) with a high weight are included in the interpolation to force the polynomial maxima within the range of 0 and 9. Furthermore, higher *m*_*i*_ values receive a higher weight to map the polynomial to the maxima. The gradient 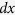 of the inflexion point (red points in Figs 3 and 5) with the smallest distance from the dominant maximum (grey points in Figs 3 and 5) describes the mitochondrial distribution. For complete mitochondrial centralisation the gradient becomes minimal (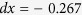; Fig. 3a). In the case of complete peripheralisation, the gradient is maximal (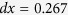; Fig. 3c) and a homogeneous MD is denoted by a 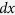 close to 0 (Fig. 3b).

#### Radial profiles analysis (RPA)

Although the AFA method works well in cells with a relatively circular shape, problems arise in cells with extremely ragged outlines or eccentric nuclei. Thus, we have developed an alternative method based on a previously published algorithm^35^, which is unaffected by these problems. In general, RPA works by generating a number of radial profiles within the cytoplasm, and subsequent analysis is performed on the grey level distribution.

In the first step, *l* equiangular radial line segments within *P*_*cytoplasm*_ are generated (Fig. 4b,h, coloured lines). Each line is then sampled at 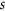 equidistant points and the bilinear interpolated grey values at these sampling points form one radial profile *p*. All profiles are stacked together to form a *s *× *l* matrix, which represents the unrolled length-normalised cytoplasm (Fig. 4e,f). To reduce the effect of non-mitochondrial pixels and background noise, each column profile *p* is thresholded separately via Otsu’s method^34^, depicted in Fig. 4c (before thresholding) and Fig. 4d (after thresholding).

For quantification of the mitochondrial distribution, the grey values of each column profile *p* are summed from the inner to the outer sampling point until 50% of the cumulative grey value of *p* is reached. The index 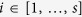 of this point is the distribution value *Distr* of one profile, and the normalised mean of all profiles 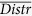 represents the mitochondrial distribution of the cell. Distinct eccentric nuclei might be indicative of tilted optical sectioning, resulting in a single-sided impaired information density. Therefore, extraordinarily long and short radial profile line outliers are filtered out using the median absolute deviation (MAD)^36^.

### Cell culture and *in vitro* measurement of cell metabolism using MPT-FLIM

The human keratinocyte cell line HaCaT was cultivated as previously reported^37^,^38^. Briefly, HaCaT cells were maintained at 37 °C under 5% CO_2_ in RPMI 1640 medium supplemented with 10% foetal calf serum, 1% L-glutamine and 1% penicillin/streptomycin. Twenty-four hours prior to the experiment, the cells were seeded into a 0.2 μm luer slide (ibidi GmbH, Munich, Germany). Multiphoton tomography was carried out 4 hours after treatment with 5 μM rotenone or 20 mM glucose (both Sigma-Aldrich, Steinheim, Germany) or 10 nM recombinant human TNF-alpha. The experiments were performed in HEPES-buffered Ringer’s solution containing (in mmol/L): 140 NaCl, 5 KCl, 1 MgCl, 1 CaCl_2_, 5 glucose, and 10 HEPES (N-2-hydroxyethylpiperazin-N0-2-ethanesulfonic acid), adjusted to a pH of 7.4, at 37 °C.

### Preparation and analysis of *in vivo* MPT-FLIM data

Because of the vast amount of obtained image and FLIM data, a specialised system for structured data storage, retrieval and analysis was needed. For this purpose, we used the Open Microscopy Environment (OME). OME consists of OME-XML^27^, an open standard format for the description and annotation of microscopy image data, and OMERO (OME Remote Objects)^39^, an open source software for the management and analysis of image data.

To enrich the existing image data with histological parameters, all images were first categorised by a dermatologist with expertise in the field of histopathology. For categorisation, a region of interest consisting only of cells from one single epidermal layer was defined for each image. Additional examples of parameters are the image quality with regard to suitability for automatic analysis and diagnosis from a histological perspective. Categorisation parameters, clinical values (e.g., medication and the SCORAD) and technical image acquisition data (e.g., excitation wavelength and laser energy) were transformed into the OME-XML format, merged with the associated image and FLIM data and then imported to the OMERO server. Various clients (OMERO.insight, OMERO.web) can be used for manual image and metadata browsing. Furthermore, the functionality of the OMERO server can be used and extended to implement algorithms for analysis. To obtain a tool for specialised and explorative data analysis and quantification, we have chosen a two-staged approach. In the first step, the user creates a subset of all images by defining various metadata constraints (e.g., image quality: high to very high; epidermal layers: *Stratum granulosum* and *spinosum*, and SCORAD: 10 to 50), and in a second step, the parameters for quantitative analysis (e.g., SCOARD vs. tau_m_) are chosen. For rapid evaluation of results, different visualisation methods, such as box or scatter plots, are available, and the quantitative results can be exported for further analysis.

### Statistical computation

Mean data from the experiments are presented with the standard error of the mean (SEM). Statistical computations were performed with SAS 9.2 (SAS Institute Inc., Cary, NC, USA).

## Additional Information

**How to cite this article**: Huck, V. *et al*. From morphology to biochemical state – intravital multiphoton fluorescence lifetime imaging of inflamed human skin. *Sci. Rep*. **6**, 22789; doi: 10.1038/srep22789 (2016).

## Supplementary Material

Supplementary Information

Supplementary Movie S3

## References

[b1] Bieber T. Atopic dermatitis. N. Engl. J. Med. 358, 1483–1494 (2008). 18385500 10.1056/NEJMra074081

[b2] Cork M. J. . New perspectives on epidermal barrier dysfunction in atopic dermatitis: gene-environment interactions. J. Allergy Clin. Immunol. 118, 3–21; quiz 22–23 (2006). 10.1016/j.jaci.2006.04.042 16815133

[b3] Becker W. . Fluorescence lifetime imaging by time-correlated single-photon counting. Microsc. Res. Tech. 63, 58–66 (2004). 14677134 10.1002/jemt.10421

[b4] Koehler M. J. . Clinical application of multiphoton tomography in combination with confocal laser scanning microscopy for *in vivo* evaluation of skin diseases. Exp. Dermatol. 20, 589–594 (2011). 21539618 10.1111/j.1600-0625.2011.01279.x

[b5] Koenig K. & Riemann I. High-resolution multiphoton tomography of human skin with subcellular spatial resolution and picosecond time resolution. J Biomed Opt 8, 432–439 (2003). 12880349 10.1117/1.1577349

[b6] Williams R. M., Zipfel W. R. & Webb W. W. Multiphoton microscopy in biological research. Curr. Opin. Chem. Biol. 5, 603–608 (2001). 11578936 10.1016/s1367-5931(00)00241-6

[b7] Fischer F. . Assessing the risk of skin damage due to femtosecond laser irradiation. J Biophotonics 1, 470–477 (2008). 19343673 10.1002/jbio.200810050

[b8] Kable E. P. & Kiemer A. K. Non-invasive live-cell measurement of changes in macrophage NAD(P)H by two-photon microscopy. Immunol. Lett. 96, 33–38 (2005). 15585305 10.1016/j.imlet.2003.12.013

[b9] Lakowicz J. R., Szmacinski H., Nowaczyk K. & Johnson M. L. Fluorescence lifetime imaging of free and protein-bound NADH. Proc. Natl. Acad. Sci. USA 89, 1271–1275 (1992). 1741380 10.1073/pnas.89.4.1271 PMC48431

[b10] Schneckenburger H., Koenig K., Kunzi-Rapp K., Westphal-Frosch C. & Ruck A. Time-resolved *in-vivo* fluorescence of photosensitizing porphyrins. J. Photochem. Photobiol. B. 21, 143–147 (1993). 8301410 10.1016/1011-1344(93)80176-a

[b11] Skala M. C. . *In vivo* multiphoton microscopy of NADH and FAD redox states, fluorescence lifetimes, and cellular morphology in precancerous epithelia. Proc. Natl. Acad. Sci. USA 104, 19494–19499 (2007). 18042710 10.1073/pnas.0708425104 PMC2148317

[b12] Patalay R. . Multiphoton multispectral fluorescence lifetime tomography for the evaluation of basal cell carcinomas. PLoS One 7, e43460 (2012). 22984428 10.1371/journal.pone.0043460 PMC3439453

[b13] Seidenari S. . Multiphoton laser tomography and fluorescence lifetime imaging of basal cell carcinoma: morphologic features for non-invasive diagnostics. Exp. Dermatol. 21, 831–836 (2012). 22882324 10.1111/j.1600-0625.2012.01554.x

[b14] Ulrich M. . *In vivo* detection of basal cell carcinoma: comparison of a reflectance confocal microscope and a multiphoton tomograph. J Biomed Opt 18, 61229 (2013). 23456144 10.1117/1.JBO.18.6.061229

[b15] Paul R. J. & Schneckenburger H. Oxygen concentration and the oxidation-reduction state of yeast: determination of free/bound NADH and flavins by time-resolved spectroscopy. Naturwissenschaften 83, 32–35 (1996). 8637605 10.1007/BF01139308

[b16] Chia T. H., Williamson A., Spencer D. D. & Levene M. J. Multiphoton fluorescence lifetime imaging of intrinsic fluorescence in human and rat brain tissue reveals spatially distinct NADH binding. Opt Express 16, 4237–4249 (2008). 18542519 10.1364/oe.16.004237

[b17] Niesner R. . Selective detection of NADPH oxidase in polymorphonuclear cells by means of NAD(P)H-based fluorescence lifetime imaging. J Biophys 2008, 602639 (2008). 20107577 10.1155/2008/602639 PMC2809359

[b18] Su G. C., Wei Y. H. & Wang H. W. NADH fluorescence as a photobiological metric in 5-aminolevlinic acid (ALA)-photodynamic therapy. Optics express 19, 21145–21154 (2011). 22108965 10.1364/OE.19.021145

[b19] Vishwasrao H. D., Heikal A. A., Kasischke K. A. & Webb W. W. Conformational dependence of intracellular NADH on metabolic state revealed by associated fluorescence anisotropy. The Journal of biological chemistry 280, 25119–25126 (2005). 15863500 10.1074/jbc.M502475200

[b20] Seidenari S. . Multiphoton laser microscopy and fluorescence lifetime imaging for the evaluation of the skin. Dermatol Res Pract 2012, 810749 (2012). 22203841 10.1155/2012/810749 PMC3235701

[b21] Koenig K. Robert Feulgen Prize Lecture. Laser tweezers and multiphoton microscopes in life sciences. Histochem. Cell Biol. 114, 79–92 (2000). 11052257 10.1007/s004180000179

[b22] Heikal A. A. Intracellular coenzymes as natural biomarkers for metabolic activities and mitochondrial anomalies. Biomark Med 4, 241–263 (2010). 20406068 10.2217/bmm.10.1 PMC2905054

[b23] Chance B. Pyridine nucleotide as an indicator of the oxygen requirements for energy-linked functions of mitochondria. Circ. Res. 38, I31–38 (1976). 178460

[b24] Bito T. & Nishigori C. Impact of reactive oxygen species on keratinocyte signaling pathways. J. Dermatol. Sci. 68, 3–8 (2012). 22771322 10.1016/j.jdermsci.2012.06.006

[b25] Gros E., Bussmann C., Bieber T., Forster I. & Novak N. Expression of chemokines and chemokine receptors in lesional and nonlesional upper skin of patients with atopic dermatitis. J. Allergy Clin. Immunol. 124, 753–760 e751 (2009). 19767072 10.1016/j.jaci.2009.07.004

[b26] Suarez-Farinas M. . Nonlesional atopic dermatitis skin is characterized by broad terminal differentiation defects and variable immune abnormalities. J. Allergy Clin. Immunol. 127, 954–964 e951–954 (2011). 21388663 10.1016/j.jaci.2010.12.1124 PMC3128983

[b27] Linkert M. . Metadata matters: access to image data in the real world. J. Cell Biol. 189, 777–782 (2010). 20513764 10.1083/jcb.201004104 PMC2878938

[b28] Severity scoring of atopic dermatitis: the SCORAD index. Consensus Report of the European Task Force on Atopic Dermatitis. Dermatology 186, 23–31 (1993). 8435513 10.1159/000247298

[b29] Rickham P. P. Human Experimentation. Code of Ethics of the World Medical Association. Declaration of Helsinki. Br. Med. J. 2, 177 (1964). 14150898 10.1136/bmj.2.5402.177 PMC1816102

[b30] *The International Conference on Harmonisation of Technical Requirements for Registration of Pharmaceuticals for Human Use*. (2015) Available at: http://www.ich.org. (Accessed: 15th May 2015). 10.1111/j.1365-2125.1994.tb05705.x PMC1364893 8054244

[b31] McAuliffe M. J. . In Computer-Based Medical Systems, 2001. CBMS 2001. Proceedings. 14th IEEE Symposium on. 381–386.

[b32] Gonzalez R. C. & Woods R. E. In Digital image processing 3rd edn, 100–101 (Prentice Hall, 2008).

[b33] Leong F. J., Brady M. & McGee J. O. Correction of uneven illumination (vignetting) in digital microscopy images. J. Clin. Pathol. 56, 619–621 (2003). 12890815 10.1136/jcp.56.8.619 PMC1770032

[b34] Otsu N. Threshold Selection Method from Gray-Level Histograms. Ieee T Syst Man Cyb 9, 62–66 (1979).

[b35] Sanches J. M. . Quantification of mutant E-cadherin using bioimaging analysis of *in situ* fluorescence microscopy. A new approach to CDH1 missense variants. Eur. J. Hum. Genet. 23, 1072–1079 (2015). 25388006 10.1038/ejhg.2014.240 PMC4795115

[b36] Leys C., Ley C., Klein O., Bernard P. & Licata L. Detecting outliers: Do not use standard deviation around the mean, use absolute deviation around the median. J. Exp. Soc. Psychol. 49, 764–766 (2013).

[b37] Boukamp P. . Normal keratinization in a spontaneously immortalized aneuploid human keratinocyte cell line. J. Cell Biol. 106, 761–771 (1988). 2450098 10.1083/jcb.106.3.761 PMC2115116

[b38] Schulz J. . The amido black assay: a simple and quantitative multipurpose test of adhesion, proliferation, and cytotoxicity in microplate cultures of keratinocytes (HaCaT) and other cell types growing adherently or in suspension. J. Immunol. Methods 167, 1–13 (1994). 7508474 10.1016/0022-1759(94)90069-8

[b39] Swedlow J. R. & Eliceiri K. W. Open source bioimage informatics for cell biology. Trends Cell Biol. 19, 656–660 (2009). 19833518 10.1016/j.tcb.2009.08.007 PMC2789254

