# Supplementary material for: From morphology to biochemical state – intravital multiphoton fluorescence lifetime imaging of inflamed human skin
Source: Sci Rep. 2016 Mar 23;6:22789. doi: 10.1038/srep22789 (PMC4804294; doi:10.1038/srep22789)
Supplement: Supplementary Information [file srep22789-s1.pdf]

Supplementary Information

From morphology to biochemical state – intravital multiphoton fluorescence lifetime imaging of inflamed human skin

Volker Huck[1], Christian Gorzelanny[1], Kai Thomas[2], Valentina Getova[2], Verena Niemeyer[1], Katharina Zens[1], Tim R. Unnerstall[2], Julia S. Feger[1], Mohammad A. Fallah[3] Dieter Metze[2], Sonja Ständer[2], Thomas A. Luger[2], Karsten Koenig[4,5], Christian Mess[1,2,*], and Stefan W. Schneider[1,*]

[1] Heidelberg University, Medical Faculty Mannheim, Experimental Dermatology, Theodor-Kutzer-Ufer 1-3, 68167 Mannheim, Germany.

[2] University of Münster, Department of Dermatology, Von-Esmarch-Str. 58, 48149 Münster, Germany.

[3] University of Konstanz, Department of Biophysical Chemistry, Universitätsstr. 10, 78457 Konstanz, Germany.

[4] Saarland University, Mechatronics & Physics, Campus A5 1, 66123 Saarbrücken, Germany.

[5] JenLab GmbH, Schillerstr. 1, 07745 Jena, Germany.

* Corresponding authors

Supplementary Figures

Supplementary Figure S1.

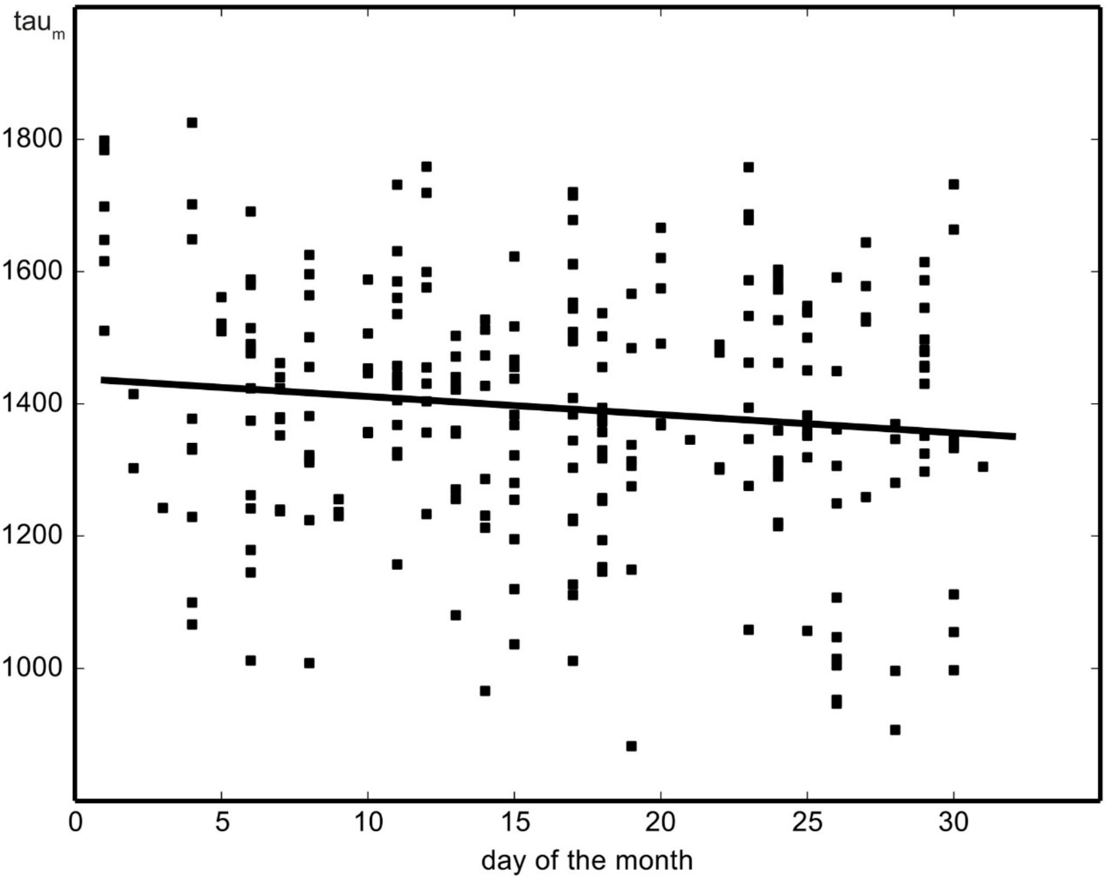

Control correlation to evaluate the reliability of the study design.

Correlation between the day of the month of the examination and the tau_m value.

Supplementary Figure S2.

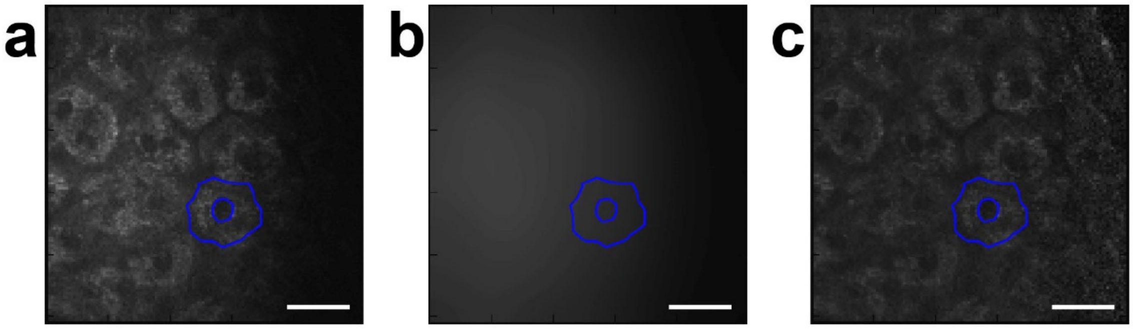

Shading correction.

(**a**) A *Stratum granulosum* greyscale image without corrections and a segmented cell marked in blue. The vignetting effect is visible in the right image area. (**b**) The image after Gaussian filtering (σ = 40). (**c**) The corrected image after the pixel-wise division of (**a**) by (**b**). The vignetting effect is removed. Scale bars correspond to 20 µm.

Supplementary Methods

Shading correction

Some segmented cells suffer from shading deficiencies due to systemic irregular illumination at the periphery (vignetting) or to a non-optimal sectional plane (Supplementary Fig. S2 a). To address this issue, we utilise a shading correction method based on Gaussian filtering. The continuous Gaussian function for two dimensions is defined as

$$f(x,y) = \frac{1}{2\pi\sigma^2} \cdot e^{-\frac{x^2+y^2}{2\sigma^2}}$$

with σ denoting the standard deviation that controls the strength of the smoothing effect. The photon count image I (Supplementary Fig. S2 a), is convoluted by a Gaussian kernel with $\sigma = 40$ resulting in the shading image I_{Gauss} (Supplementary Fig. S2 b), which represents the general brightness distribution of I. The corrected image $I_{corr} = I \div I_{Gauss}$ (Supplementary Fig. S2 c) is then calculated via pixel-wise division and used for further processing.

Supplementary Movie Legend

Supplementary Movie S3. Three-dimensional reconstruction of a multiphoton tomographic optical biopsy of healthy human skin.

Multiphoton-based autofluorescence signals of NADH enable characterisation of the epidermal architecture with a subcellular resolution. Dermal collagen in the *Stratum papillare* is pseudocolour-coded in green. Red scale bar corresponds to 20 µm.
